# Clinicopathologic profiling and oncologic outcomes of secretory carcinoma of the breast

**DOI:** 10.1038/s41598-021-94351-w

**Published:** 2021-07-19

**Authors:** Piguo Gong, Chen Xia, Yifeng Yang, Wang Lei, Weiping Yang, Junhua Yu, Yishun Ji, Lijun Ren, Fugui Ye

**Affiliations:** 1 Department of Thyroid Breast Surgery, Qingdao Chengyang People’s Hospital, Qingdao, 266109 Shandong China; 2 Department of Medical Oncology, Hunan Cancer Hospital, The Affiliated Cancer Hospital of Xiangya School of Medicine, Central South University, Changsha, 410013 China; 3 Department of Breast Surgery, Affiliated Hospital of Jining Medical University, Jining, 27200 Shandong China; 4 Key Laboratory of Breast Cancer in Shanghai, Department of Breast Surgery, Fudan University Shanghai Cancer Center, Shanghai, 200032 China

**Keywords:** Medical research, Oncology

## Abstract

Secretory carcinoma of the breast (SCB) is a rather rare entity of invasive breast cancer, the clinicopathologic characteristics and survival outcomes remain to be elaborated. A retrospective review was conducted in SEER database. A total of 190 SCB patients identified in SEER were eligible for inclusion in the analysis. Median age at diagnosis was 56 years (range 2–96 years). Both sexes and bilateral breast could be affected. Intriguingly, the incidence of SCB tended towards to decreasing in recent decades. Small tumor burden was observed with a mean tumor size of 2.13 cm. In a subgroup with sufficient details, positive staining of estrogen receptor (ER) and progesterone receptor (PR) was 58% and 40%, respectively. The vast majority of patients were of well to moderate differentiation (86.86%) and negative regional lymph nodes involvement (70.71%). Nearly half of the patients took radiotherapy and chemotherapy. Seniors were inclined to have an inferior breast cancer specific survival (BCSS) than their younger counterparts (P = 0.018). Patients underwent breast conserving surgery (BCS) and radiotherapy had much better BCSS than its mastectomy counterparts (P = 0.014). Collectively, SCB is a clinical indolent invasive breast cancer with excellent prognosis. BCS in conjunction with radiotherapy would be a rational alternative for this distinct entity.

## Introduction

Literature reported that rare cancer constitutes more than 20% of all cancer diagnoses annually^1^. To date, limited studies have been conducted on rare cancers, therefore many aspects are blind to clinicians, causing a worse prognosis for patients with rare cancers in comparison with common cancers. Another dilemma is that little is known regarding approaches to prevent and accurate diagnosis of many rare cancers^2^. Recent decades, tremendous advances in cancer genomics and cancer biology have resulted in great progress in cancer treatment profiles, leading to entrance the era of individualized precision medicine^3^. Consequently, focusing on the extremely rare variants of cancer would be of remarkable significance to improve the recognition and survival outcomes of the entire cancer community.

Secretory carcinoma of the breast (SCB) is a scarce but distinct subtype of breast malignancy, initially known as juvenile breast carcinoma by McDivitt and Stewart in 1966^4^, accounting for less than 0.15% of all breast cancers^5^. Usually, SCB has been reported as triple negative immunophenotype and positive staining for cytokeratins and EGFR, parallel to basal-like breast cancer, while the indolent clinical course as well as prolonged survival seem opposite to common triple negative breast cancer (TNBC)^6–8^. Of note, few studies demonstrated that SCB could present with positivity for hormone receptors^9–11^. Cytogenetically, ETV6-NTRK3 gene fusion is deemed as the most characteristic feature of genomic alteration of SBC^12,13^. The ETV6-NTRK3 protein with transforming activity, could leads to constitutive activation of Ras-MAP kinase and phosphatidyl inositol-3-kinase-AKT pathways, participating in SCB carcinogenesis^12^. Thus, targeting ETV6-NTRK3 has been the priority of biomedical investigation. Given the exceeding rarity of SCB, there is not consensus regarding the optimal diagnostic criterium, treatment strategies and to what extent the excellent prognosis could be ascribed to clinicopathologic parameters.

The large data volume in Survival, Epidemiology and End Results (SEER) program provides a well-established platform to investigate rare tumors. In this study, to help address the existing discrepancies and deep understand the nature of SCB, we examine the clinical and pathologic characteristics and long-term survival outcomes of SCB in a large number of cases. The findings probably shed light on our better understanding and management of SCB.

## Results

### Patient demographics

**Figure 1:**
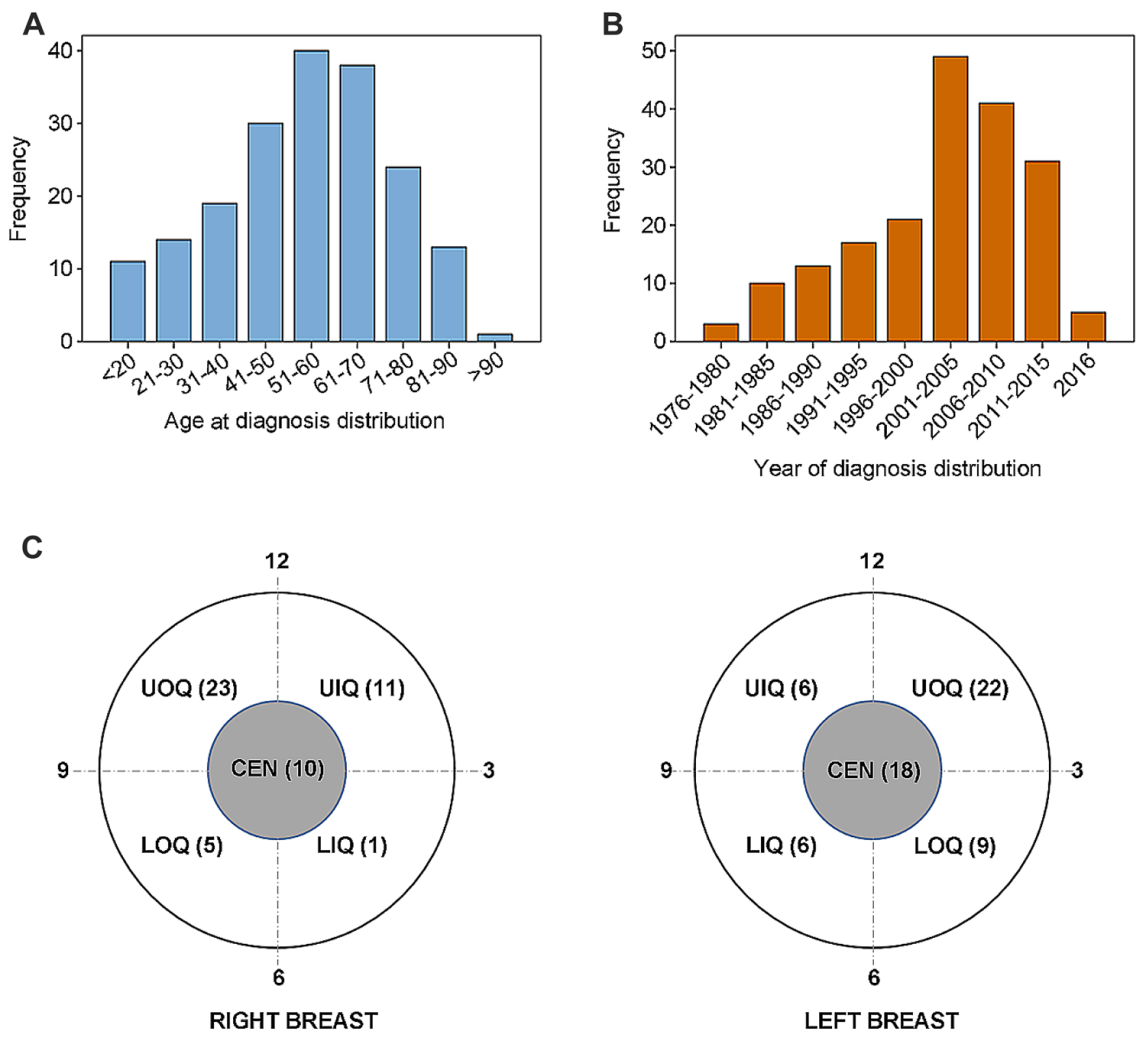
Clinicopathologic characteristics of study cohort. (**A**) Distribution of age at diagnosis. (**B**) Distribution of year of diagnosis. (**C**) Distribution of tumor location in the breasts. *UIQ* upper inner quadrant, *LIQ* lower inner quadrant, *LOQ* lower outer quadrant, *UOQ* upper outer quadrant, *CEN* nipple-areola complex.

The demographic characteristics of the 190 SCB patients was summarized in Fig. 1. Median age at diagnosis was 56 years (range 2–96 years) (data not shown). Both sexes could be affected by SCB, with an approximate male to female ratio of 1:30 (data not shown). The proportion of patients diagnosed under the age of 30 years was 13.16% (Fig. 1A). Intriguingly, the number of patients was stable increasing as time went on, with a peak occurring at the beginning of twenty-first century, while declined sharply thereafter (Fig. 1B). Laterality was found to be comparable between the left and right breast, with 103 patients in left, 85 in right and 2 bilateral, respectively (data not shown). Primary tumor site was highest likely located in the upper-outer quadrant (UOQ) irrespective of laterality (Fig. 1C).

### Disease and treatment characteristics

In order to better understand the clinicopathologic features of SCB, a subgroup of 99 patients with sufficient and explicit information were derived and listed in Table 1. Median age at diagnosis was 57 years (range: 8–89 years). Most patients were female white ethnicity and had tumors no more than 2 cm (cm), with an average tumor size of 2.13 cm. The vast majority of patients were of negative regional lymph nodes involvement (70.71%) and well to moderate differentiation (86.86%). Positive staining of estrogen receptor (ER) and progesterone receptor (PR) was 58% and 40%, respectively. BCS was performed in 53 patients. The uptake of radiotherapy and chemotherapy was 45.45% and 41.41%, respectively. Hormone therapy was unavailable in SEER database.

**Table 1:** Clinicopathologic characteristics and type of treatment of all patients included.

Characteristics	All patients (n = 99)	
	No.	%
**Age at diagnosis (years)**		
Median	57	
Range (IQR)	45–66	
**Gender**		
Female	95	95.96
Male	4	4.04
**Laterality**		
Left	55	55.56
Right	44	44.44
**Ethnicity**		
White	82	82.83
Black	11	11.11
Other	6	6.06
**Pathologic tumor size**		
pT1	64	64.65
pT2	30	30.30
pT3	5	5.05
**Nodal status**		
pN0	70	70.71
pN1	25	25.25
pN2	4	4.04
**Nuclear grade**		
I	41	41.41
II	45	45.45
III	13	13.13
**ER status**		
Positive	58	58.59
Negative	41	41.41
**PR status**		
Positive	40	40.40
Negative	59	59.60
**Surgery modality**		
BCS	53	53.54
Mastectomy	46	46.46
**Radiation therapy**		
Yes	45	45.45
No	54	54.55
**Chemotherapy**		
Yes	41	41.41
No/unknown	58	58.59

*pT1* pathological tumor size ≤ 2 cm, *pT2* 2 cm < pathological tumor size ≤ 5 cm, *pT3* pathological tumor size > 5 cm, *pN0* negative regional lymph node, *pN1*, 1 to 3 regional lymph node metastasis, *pN2* 4 to 9 regional lymph node metastasis, *Nuclear grade I* well differentiation, *Nuclear grade II* moderate differentiation, *Nuclear grade III* poor differentiation, *ER* estrogen receptor, *PR* progesterone receptor, *BCS* breast conserving surgery.

### Oncologic outcomes

**Figure 2:**
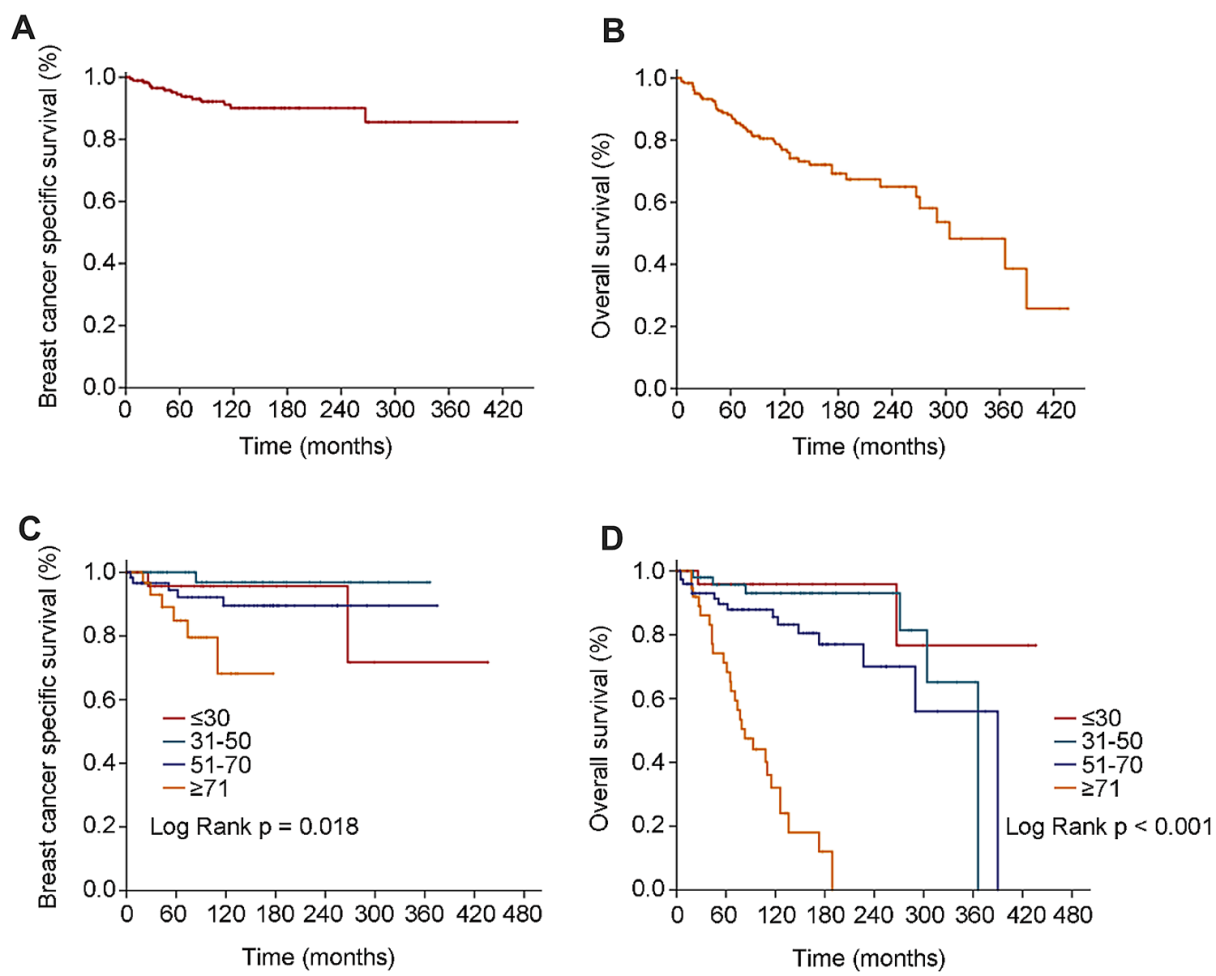
OS (overall survival) and BCSS (breast cancer specific survival) of study cohort. (**A**) Kaplan and Meier estimates BCSS. (**B**) Kaplan and Meier estimates of OS. (**C**) Kaplan and Meier estimates of age-related BCSS. (**D**) Kaplan and Meier estimates of age-related OS.

For all patients, the median follow-up time was 97 months (range 0–436 months). 5-year BCSS was 95.79%, 10-year and 20-year BCSS both were 93.16% (Fig. 2A). 5-year OS was 89.47%, 10-year OS was 81.58% and 20-year OS was 76.84% (Fig. 2B). Age-specific survivals were presented in (Fig. 2C,D). There was statistical significance in age-specific BCSS (P = 0.018) and OS (P < 0.001), and worse prognosis was indicated with growing age.

**Figure 3:**
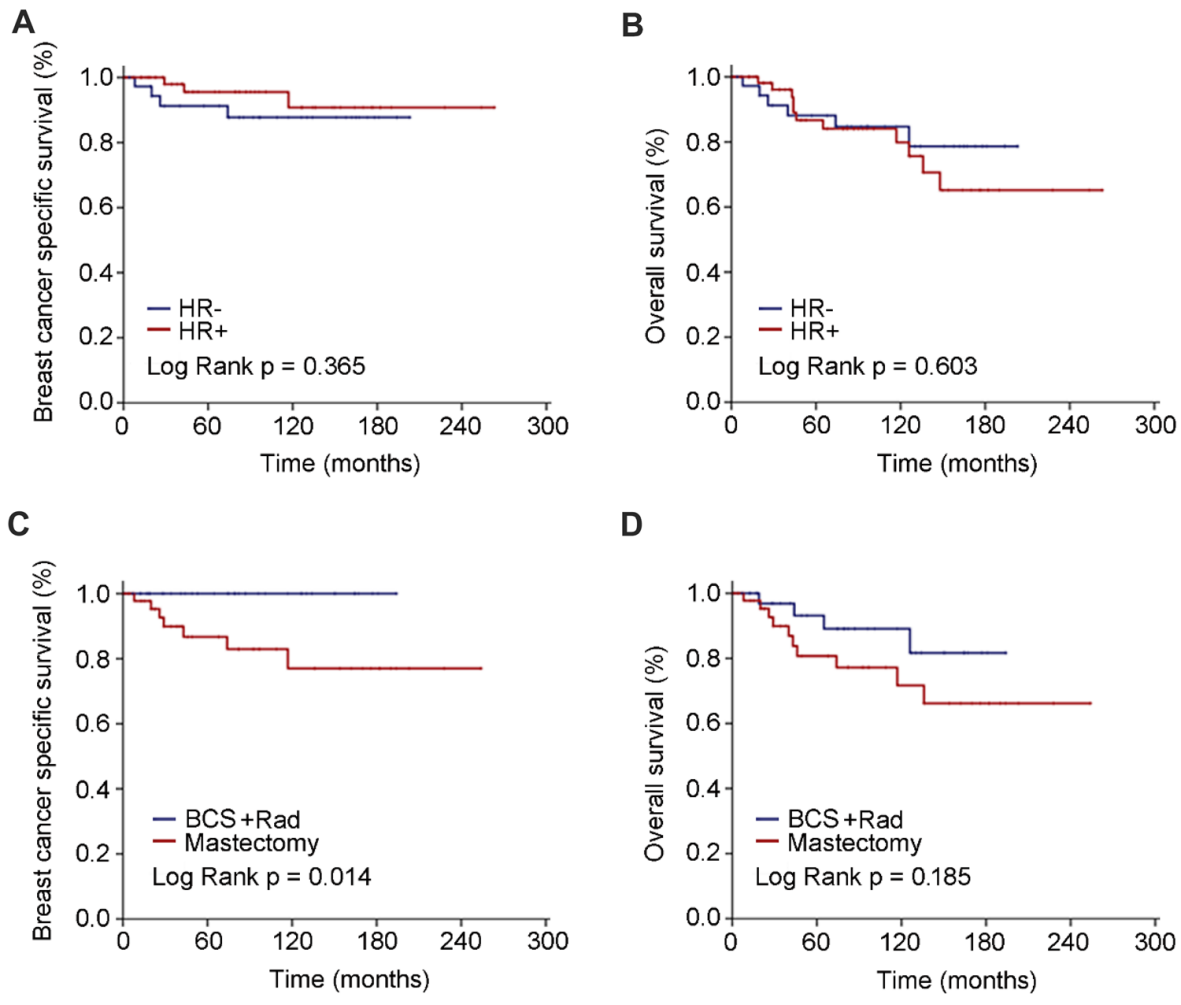
Survival outcomes categorized by hormone receptor and surgery modalities. (**A**) Kaplan and Meier estimates of BCSS (breast cancer specific survival) grouped by HR (hormone receptor) status. (**B**) Kaplan and Meier estimates of OS (overall survival) grouped by HR status. (**C**) Kaplan and Meier estimates of BCSS grouped by BCS (breast conserving surgery) and radiotherapy and mastectomy. (**D**) Kaplan and Meier estimates of OS grouped by BCS and radiotherapy and mastectomy.

For the subgroup cohort, the median follow-up time was 82 months (range 0-263 months). No statistical significance was observed with regard to BCSS (P = 0.365) and OS (P = 0.603) categorized by hormone receptor status (Fig. 3A,B). Similar baseline characteristics of patients underwent BCS and radiotherapy and mastectomy were shown in Table 2. Although OS (P = 0.185) was comparable between the two subsets, patients who underwent BCS and radiotherapy had much better BCSS than its mastectomy counterparts (P = 0.014) (Fig. 3C,D).

**Table 2:** Clinicopathologic characteristics and chemotherapy of subgroups divided by type of surgery.

Characteristics	BCS + Rad (n = 37)		Mastectomy (n = 46)		*p*-value
	No	%	No	%	
**Age at diagnosis (years)**					
Median	58		57		
Range (IQR)	48–66		41–67		
**Gender**					0.125
Female	37	100.00	42	91.30	
Male	0	0.00	4	8.70	
**Laterality**					0.840
Left	20	54.05	27	58.70	
Right	17	45.95	19	41.30	
**Ethnicity**					0.330
White	32	86.49	37	80.43	
Black	2	5.41	7	15.22	
Other	3	8.11	2	4.35	
**Pathologic tumor size**					0.052
pT1	29	78.38	25	54.35	
pT2	6	16.22	18	39.13	
pT3	2	5.41	3	6.52	
**Nodal status**					0.722
pN0	28	75.68	29	63.04	
pN1	7	18.92	15	32.61	
pN2	2	5.41	2	4.35	
**Nuclear grade**					0.527
I	17	45.95	17	36.96	
II	17	45.95	22	47.83	
III	3	8.11	7	15.22	
**ER status**					0.729
Positive	24	64.86	27	58.70	
Negative	13	35.14	19	41.30	
**PR status**					0.420
Positive	17	45.95	16	34.78	
Negative	20	54.05	30	65.22	
**Chemotherapy**					0.998
Yes	17	45.95	20	43.48	
No/unknown	20	54.05	26	56.52	

*pT1* pathological tumor size ≤ 2 cm, *pT2* 2 cm < pathological tumor size ≤ 5 cm, *pT3* pathological tumor size > 5 cm, *pN0* negative regional lymph node, *pN1*, 1 to 3 regional lymph node metastasis, *pN2* 4 to 9 regional lymph node metastasis, *Nuclear grade I* well differentiation, *Nuclear grade II* moderate differentiation, *Nuclear grade III* poor differentiation, *ER* estrogen receptor, *PR* progesterone receptor, *BCS* breast conserving surgery.

## Discussion

SCB is a rare subtype of invasive breast malignancy, previous studies have been largely limited by case reports and small case series^5^. In the present study, we examined the demographics, disease characteristics, patterns of treatment and survival outcomes for patients diagnosed with SCB in a large-sized cohort derived from SEER database.

Despite initially discovered in juveniles, it was documented in a wide range of age groups and more frequently occur in adults later^14^. Till now, the two largest retrospective studies conducted by Horowitz et al. with 83 SCB patients published in 2012^15^. and Jacob et al. with 246 SCB patients published in 2016^5^ had illustrated a median age at diagnosis of 53 years in SEER database and 56 years in the National Cancer Data Base. Consistent with prior studies, median age at diagnosis was 56 years in this study. Additionally, Jacob et al.^5^ demonstrated a male to female ratio of 1:31, which was in agreement with that of 1:30 in our study. Nevertheless, a published experience of male to female ratio was approximately 1:6^16^, which might be a result of small simple size. Confirming the previous report, the most common location of SCB was the UOQ of the breast, resembling that of invasive ductal carcinoma^17^.

Of note, great advances have been established in our understanding of biological nature of breast cancer, while the incidence of SCB was prone to continuously declining in recent decades. This was a new finding in the present study and some possible reasons would be rational to explain this phenomenon. Typically, SCB was presented with a slow-growing, painless, well-circumscribed, mobile mass, similar to that of benign epithelial proliferating lesions^8,18,19^, which may increase the possibility of misdiagnosis. Furthermore, non-specific and sparse ultrasound and mammographic findings associated with limited diagnostic value of ETV6-NTRK3 resulted in difficulty to differentiate diagnosis of SCB^17,20–22^.

There was no consistence on status of ER and PR receptor. The overwhelming majority of literature showed that SCB was negative for ER, PR, human epidermal growth factor (HER2), and positive for basal-cell markers, therefore could be classified as a peculiar subtype of TNBC^6,23,24^. Recent studies with large sample size concluded that SCB mimicked immunoprofile of hormone receptor positive cancer other than that of TNBC^5,11,25^, which was in stark contrast to the earlier reports. Our results were in support of the recent studies. In addition, other disease parameters were comparable with most previous studies, demonstrating that SCB usually presented as low grade, less likely to positive regional lymph node^26,27^.

Although axillary lymph node metastasis of SCB was described as high as approximately 15%-30% and patients with more than four lymph nodes were exclusively rare, the prognosis of SCB was excellent in published reports^8,28^. Horowitz et al.^15^ depicted that 5-year BCSS was 94.4% and 10-year BCSS was 91.4% in a study cohort of 83 patients. Recently, Li et al.^11^ uncovered that 5-year OS was 93.2%, and 10-year OS was 88.6% in a study cohort of 44 patients. Our results were in favor of prior studies, with a 5-year BCSS was 95.79%, 10-year and 20-year BCSS both were 93.16% as well as 5-year OS was 89.47%, 10-year OS was 81.58% and 20-year OS was 76.84%. To a great extent, the excellent prognosis might be contributed to the early stage and mild clinicopathologic characteristics, aside from adjuvant systemic therapy. One should be cautious that distant metastasis could occur as late as 16 years after definitive local surgery^29^. Currently, the primary management of SCB was surgery, because of the indolent clinical course and fairly good prognosis. The impact beyond surgery on survival outcomes was largely unknown. We demonstrated that BCS and radiotherapy was superior to mastectomy with comparable OS but better BCSS. Genomic study of SCB indicated that mutational burden and copy number variant of secretory carcinoma of the breast is very low^30^, which supported the BCS and radiotherapy might be sufficient to the treatment of this rare subtype.

The current study has some limitations. One limitation of this study was that details of systematic therapy, such as hormonal therapy and targeted therapy, were unavailable. Thus, the contribution of systematic therapy on the outcomes needed to be further clarified in future study. Another limitation of this study was the subtype of patients based on immunohistochemistry was not fully available, namely the frequency of receptor status as well as distribution of subtype merited deeper investigation. Although limitations existed, our study indeed improved our understanding this rare entity of SCB patients, especially based on the well-balanced characteristics of compared subgroups.

In conclusion, our retrospective study with medium-sized sample size reinforced the indolent course and unexceptionable outcomes of SCB. Moreover, BCS and radiotherapy could be a reasonable treatment of SCB followed by adequate systematic therapy. More intensive supervision and follow-up should be emphasized to the senior SCB patients. Long-term surveillance should be emphasized and cooperative work are stringently needed to derived a precise conclusion. Future studies with large size and comprehensive details are urged to validate our results.

## Methods

### Study cohort

This study adopted data from the National Cancer Institute’s Surveillance, Epidemiology, and End Results (SEER) 18 tumor registry database that was updated in November 2016. The SEER*Stat version 8.3.5 was used to case extraction. With site recode limited to breast, eligible patients were extracted based on the following inclusion criteria: pathologic diagnosis according to the International Classification of Disease for Oncology, Third Edition (ICD-O-3), one primary site only and known age at diagnosis. Consequently, a total of 190 patients with pathologically confirmed invasive secretory carcinoma of the breast (ICD-O-3 8502/3) were identified and subjected to clinicopathologic characteristics and overall prognostic analyses. Patients with unknown information of variables of laterality, pathologic tumor size, nodal status, nuclear grade, ER status, PR status and type of surgery were excluded, thereafter 99 patients left to comprise a subgroup. The influence of clinicopathologic parameters and treatment interventions on survival outcomes were performed at the subgroup setting.

### Definition of outcomes

Overall survival (OS), defined as the time from diagnosis to death from any cause. Cancer-specific survival (CSS), defined as the interval from initial diagnosis to death resulting from breast cancer.

### Statistical analysis

Survival curves were established by using the method of Kaplan and Meier. The log-rank test was adopted to compare survival outcomes between different subgroups of patients. Categorical variables were compared using the Pearson’s chi-square test or Fisher’s exact test, as appropriate. All tests were two-sided, and P < 0.05 was considered statistically significant. Statistical analysis was performed by SPSS for windows (version 23.0, SPSS Inc., Chicago, IL, USA).

### Ethics statement

This study was approved by the Ethical Committee of the Shanghai Cancer Center of Fudan University. We have submitted a request for the SEER data and complied with the sample data use agreement. The data released by the SEER database are publicly available and do not require informed patient consent. All methods were performed in accordance with relevant guidelines and regulations.
